# Implementation of cross correlation for energy discrimination on the time-of-flight spectrometer CORELLI^1^

**DOI:** 10.1107/S160057671800403X

**Published:** 2018-03-26

**Authors:** Feng Ye, Yaohua Liu, Ross Whitfield, Ray Osborn, Stephan Rosenkranz

**Affiliations:** a Neutron Scattering Division, Oak Ridge National Laboratory, Oak Ridge, TN 37830, USA; b Materials Science Division, Argonne National Laboratory, Argonne, IL 60439, USA

**Keywords:** neutron diffraction, energy discrimination, cross-correlation technique, time-of-flight neutron beamlines

## Abstract

The CORELLI instrument at Oak Ridge National Laboratory is a statistical chopper spectrometer designed and optimized to probe complex disorder in crystalline materials through diffuse scattering experiments. On CORELLI, the high efficiency of white-beam Laue diffraction combined with elastic discrimination have enabled an unprecedented data collection rate to obtain both the total and the elastic-only scattering over a large volume of reciprocal space from a single measurement. To achieve this, CORELLI is equipped with a statistical chopper to modulate the incoming neutron beam quasi-randomly, and then the cross-correlation method is applied to reconstruct the elastic component from the scattering data. Details of the implementation of the cross-correlation method on CORELLI are given and its performance is discussed.

## Abstract 2

With implementation of the cross-correlation technique at the short-pulse Spallation Neutron Source (SNS), Oak Ridge, Tennessee, USA, CORELLI obtains both the total scattering and the elastic-only scattering simultaneously from a single measurement with an unprecedented data collection rate.

## 1. Introduction   

There is growing interest in understanding materials for which disorder and nanoscale self-organization play important roles in driving their bulk properties. Notable examples include the short-range correlation of charge and orbital orders in colossal magneto­resistive manganites (Dagotto, 2005 ▸), the stripe phase in superconducting cuprates (Kivelson *et al.*, 1998 ▸), polar nano­regions in relaxor ferro­electrics (Xu *et al.*, 2006 ▸) and short-range spin fluctuations in magnetically frustrated systems (Lee *et al.*, 2002 ▸). The ability to characterize the local structures precisely and to study how they evolve under external perturbations in crystalline materials is required to obtain an understanding of these complex phenomena that emerge from simple ingredients. Neutron diffuse scattering studies are able to probe short-range fluctuations on the nanoscale and provide unique insights into material properties driven by complex disorder. Furthermore, measurements over large volumes of reciprocal space will facilitate accurate modelling of the diffuse components (Keen & Goodwin, 2015 ▸). In general, such measurements have to be performed with sufficient momentum transfer resolution to distinguish the diffuse signal from the strong Bragg peaks, although polarization analysis can be utilized to distinguish magnetic diffuse scattering from nuclear scattering (Fennell *et al.*, 2009 ▸).

A variety of experimental tools have been used to characterize the disorder in crystalline lattices. For instance, X-ray absorption fine structure (Cao *et al.*, 2002 ▸) and neutron/X-ray pair distribution function studies (Billinge *et al.*, 1996 ▸) are employed to investigate the local structure in powder samples. Other local probes, such as nuclear magnetic resonance and Mössbauer spectroscopy, provide information on the number and symmetry of defects at element-specific sites in the lattice (Lebeugle *et al.*, 2007 ▸). Systematic studies using these techniques can provide invaluable insights into the existence and nature of atomic-scale disorder. On the other hand, coherent diffuse neutron scattering from crystalline materials provides unprecedented information on the morphology of local structures and short-range correlations in crystalline samples (Nield & Keen, 2001 ▸; Welberry, 2004 ▸; Welberry & Whitfield, 2018 ▸). Diffuse scattering is related to pair correlations of atoms or magnetic moments that deviate from the average crystal or magnetic structure, respectively, both dynamically (inelastic) and statically (elastic). A major part of the dynamic contribution comes from lattice vibrations, also known as thermal diffuse scattering. Because of zero-point motion, this scattering is present at all temperatures, even in perfect single crystals. Diffuse scattering also arises from defects in the crystal structures. Since these defects do not obey translational invariance, the scattering intensity distribution does not necessarily obey the Brillouin zone symmetry of the crystal reciprocal lattice, and generally spreads over a wide range of momentum transfer *Q* [*Q* = (4π/λ)sin(θ/2), where θ is the scattering angle and λ is the wavelength of the incident radiation].

Although single-crystal neutron diffuse scattering has been studied for decades, there remain some technical challenges that have prevented it from becoming a widely used tool. Accurate modelling of defect structures requires measurements over large volumes of three-dimensional reciprocal space, with sufficient momentum transfer resolution (or polarization analysis) to distinguish diffuse components from Bragg scattering. Large volumes can now be measured with the suitable design of high-resolution white-beam time-of-flight Laue neutron diffractometers (Michels-Clark *et al.*, 2016 ▸). However, one key obstacle to taking advantage of the wide wavelength band of time-of-flight (TOF) instruments is the lack of capability to discriminate elastic diffuse scattering from vibrational and other inelastic scattering, which is often comparable in intensity. One approach to achieve energy discrimination is to utilize the cross-correlation technique, whereby the incident beam is modulated in time in a pseudo­random way. This method was intensively investigated 50 years ago, mainly for steady-state sources and beam modulation achieved with mechanical choppers (Pál *et al.*, 1968 ▸; Sköld, 1968 ▸; Von Jan & Scherm, 1970 ▸; Price & Sköld, 1970 ▸; Gompf *et al.*, 1968 ▸; Virjo, 1969 ▸), but was also considered for periodically modulated sources, such as long-pulse reactors (Matthes, 1968 ▸; Kroó *et al.*, 1968 ▸; Amadori & Hossfeld, 1972 ▸). The technique was later applied to the new generation of short-pulsed spallation neutron sources and a proof of the principle was carried out at the Intense Pulsed Neutron Source at Argonne National Laboratory (Crawford *et al.*, 1986 ▸). The possibilities and benefits of utilizing modulation of the polarization (Gordon *et al.*, 1968 ▸; Mezei & Pellionisz, 1972 ▸; Cywinski & Williams, 1984 ▸) were also discussed. While the correlation technique enables utilization of up to 50% of the incident beam spectrum, the statistical errors of the obtained scattering function are strongly correlated, making it in general inefficient, compared with traditional methods, for measuring weak inelastic signals. Modulation techniques were nevertheless implemented, in particular for diffractometry applications (Cser *et al.*, 1981 ▸; Hiismäki, 1997 ▸), and more recently the correlation method was re-evaluated for specific applications at pulsed neutron sources (Rosenkranz & Osborn, 2008 ▸; Tomiyasu *et al.*, 2012 ▸; Mezei *et al.*, 2016 ▸).

The elastic diffuse scattering diffractometer CORELLI enables measurements of large volumes of diffuse scattering with elastic discrimination by combining the efficiency of white-beam TOF Laue diffractometers with the energy discrimination provided by the cross-correlation method (Rosenkranz & Osborn, 2008 ▸), with a gain of up to two orders of magnitude in efficiency compared with standard TOF spectrometers. The instrument is located on beamline 9 at the Spallation Neutron Source (SNS) of the Oak Ridge National Laboratory and entered the user programme in 2016. A detailed description of the instrument layout and components is provided elsewhere (Ye *et al.*, 2018 ▸).

CORELLI has a direct scattering geometry with a source-to-sample distance of *L*_1_ = 20 m and a sample-to-detector distance *L*_2_ = 2.5 m. It uses ambient decoupled water as moderator and provides a neutron flux of 2 × 10^9^ n cm^−2^ eV^−1^ s^−1^ MW^−1^ at the sample position for 50 meV incident energy. In the typical operation mode, an incident neutron energy range of 10–200 meV enables measurements over a wide momentum transfer range from 0.5 to 12 Å^−1^, with in-plane detector coverage from −18 to 148° and out-of-plane coverage from −26.5 to 29.5°. The wide detector coverage (a solid angle of approximately 2 steradian) makes the instrument highly efficient for collecting data over a large volume of reciprocal space at an unprecedented rate, and enables studies of *in situ* structural evolution in an increasingly diverse array of complex sample environments. By operating the correlation chopper asynchronously from the pulsed neutron source, all incident wavelengths are measured in a single run, and saving the total TOF as well as the current chopper phase for each detected neutron allows the elastic scattering signal to be reconstructed using the cross correlation. Simply ignoring the chopper phase, on the other hand, provides data equivalent to a standard TOF Laue diffractometer. Therefore, CORELLI provides both the purely elastic and total scattering signals simultaneously from a single experiment. In the following, we discuss in detail the implementation of the cross-correlation method at CORELLI and illustrate its performance.

**Figure 1:**
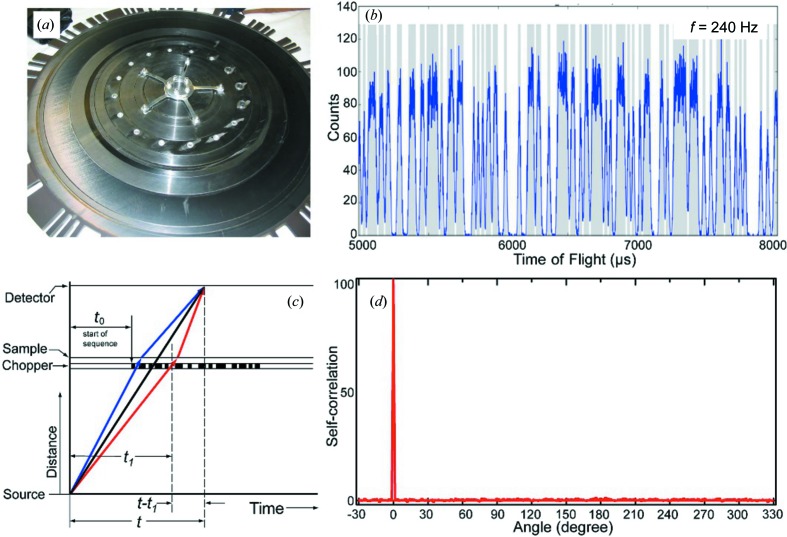
(*a*) The correlation chopper on CORELLI, made of carbon fibre and consisting of *N* = 255 open/closed elements, arranged in a pseudorandom sequence. (*b*) The modulated incident neutron spectrum, characterized by the beam monitor immediately behind the correlation chopper running at 240 Hz that is in synchronization with the master pulse of the accelerator at constant phase offset *t*_0_. The grey areas indicate the TOF windows where the chopper is in the open position, and the blue curve is the measured neutron counts. (*c*) Schematic time–distance diagram, illustrating how neutrons with different incident energies pass through the correlation chopper, are scattered at the sample position with different energy transfers and arrive at the same time at the detector. *t*_1_ specifies the time of flight that incident neutrons travel from the source to the correlation chopper, *t* is the total time of flight that neutrons travel from the source to the detector, and *t*_0_ denotes the phase offset between the first element of the sequence and the start of the neutron pulse at the source. The black line represents the elastic scattering process, whereas the other lines show scattering events that involve energy transfer and therefore a change in the neutron velocity. (*d*) The self-correlation of the actual correlation chopper is approximately a δ function.

Fig. 1 ▸ describes the principle of the unique component of the CORELLI spectrometer: the correlation chopper. The chopper is made of carbon fibre and is capable of operation at speeds of up to 18 000 r min^−1^ to achieve the desired energy resolution. The chopper has an outer diameter of 70 cm and consists of 255 elements arranged in a pseudorandom sequence (Fig. 1 ▸ *a*). This sequence has the self-correlation (SC) property of
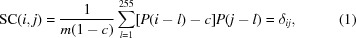
where *P*(*i*) is the transmission of the individual *i*th element of the correlation chopper, idealized as a discrete sequence with values of either 1 (open) or 0 (closed), *m* = 127 is the number of open elements, and *c* = (*m* − 1)/(*N* − 1) is the duty cycle ∼0.5, *N* = 255 being the total number of segments. When the correlation chopper is operated in synchronization with the source frequency, a repeating neutron beam modulation sequence is achieved. This is illustrated in Fig. 1 ▸(*b*), where the pulsed neutron source is operated at 60 Hz and the correlation chopper is running at 240 Hz, with a constant offset between the source pulse and the start of the correlation sequence *t*_0_. However, in normal operation mode the correlation chopper runs asynchronously from the source pulse of the accelerator, therefore providing, over the span of several source pulses, a full white spectrum of incident energies with equal average transmission probability for each energy.

**Figure 2:**
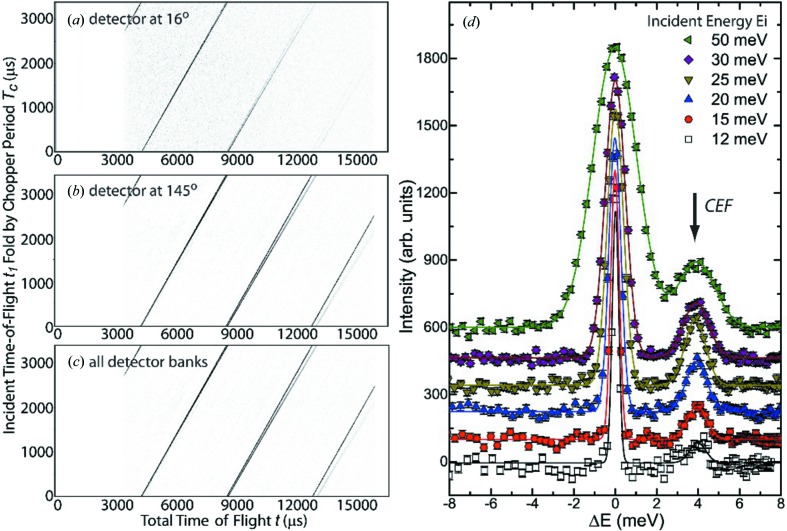
(*a*)–(*c*) The reconstructed scattering intensity [proportional to *S*(*q*, ω)] for data collected at scattering angles 2θ = 16 and 145°, and summation over all detector banks, showing the coexistence of strong elastic and weak inelastic lines. The correlation chopper is running at a frequency *f* = 293.4118 Hz (periodicity *T*_C_ = 3408 µs). The neutron events collected at the detector have a periodicity of (22.5/18)*T*_C_ = 4260 µs, where 22.5 is the distance from the moderator to the detector (in metres) and 18 is the distance from the moderator to the correlation chopper (in metres). The incident time of flight *t*_1_ [*y* axis in panels (*a*)–(*c*)] has been folded by the chopper period *T*_C_, leaving four repetition windows in the plot. Note that the elastic line goes through the origin (*t*_1_ = 0, *t* = 0). The data were from a PrAl_3_ powder sample (mass of 4 g) at 5 K. (*d*) Horizontal line cuts performed for the data in panel (*c*), with the *x*-axis units converted to energy transfer (Δ*E*), *i.e.* the line cuts in panel (*d*) with *E*_i_ = 12 and 50 meV are converted from the data in panel (*c*) near total time of flight *t* = 14 851 and 7275 µs, respectively. The CEF excitation at Δ*E* = 4 meV of PrAl_3_ is clearly resolved for all incident neutron energies between 12 and 50 meV. For clarity, the data at 15, 20, 25, 30 and 50 meV have been shifted vertically by 120, 240, 360, 480 and 600, respectively.

Because of the many chopper elements, for each chopper phase *t*_0_ the intensity measured at the detector at a specific total TOF *t* is the integration over all scattering events with probability *S*(*t*_1_, *t*), for neutrons passing through the chopper at time *t*_1_, as illustrated in Fig. 1 ▸(*c*):
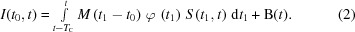
Here, *S*(*t*_1_, *t*) is the scattering function to be determined for the incident TOF *t*_1_ (the time it takes neutrons to travel from the source to the correlation chopper, related to the incident neutron energy *E*_i_) and total TOF *t* (the time it takes neutrons to travel from the source to the detector). 1/*T*_C_ is the running frequency of the correlation chopper. *M*(*t*_1_ − *t*_0_) is the actual transmission encountered when incident neutrons arrive at the chopper at *t*_1_ and the chopper has phase *t*_0_. φ(*t*_1_) is the neutron flux for the incident energy *E*_i_ corresponding to *t*_1_, and B(*t*) is the instrument background uncorrelated with the chopper phase. Since the correlation chopper rotates continuously, the actual transmission *M*(*t*_1_) has a trapezoidal shape, instead of the stepwise profile employed for *P*(*i*) in equation (1). But, as shown in Fig. 1 ▸(*d*), the self-correlation property of the pseudo­random modulation sequence remains a δ-function-like autocorrelation when defined by integration over time for the actual chopper:
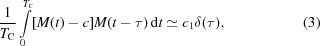
where *c*_1_ = *m*(1 − *c*)/255. The experimental data are saved in an event-based manner in the NeXus format (Könnecke *et al.*, 2015 ▸), whereby the phase of the correlation chopper is recorded as a function of absolute time in the meta-information part of the data file. This enables the determination of the phase offset *t*_0_ and TOF *t*. Once neutron events are collected with sufficient counting statistics for all different phase offsets *t*_0_, the cross correlation *C*(*t*_1_, *t*) of the scattering intensity *I*(*t*_0_, *t*) with the chopper modulation sequence *M*(*t*_1_ − *t*_0_) provides a quantity proportional to the scattering function, with an additional background term:
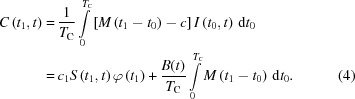



To demonstrate that the CORELLI instrument provides energy discrimination, we have measured the crystal electric field (CEF) excitation in PrAl_3_ at 5 K using a powder sample, with the temperature regulated using a closed-cycle refrigerator. The first excited CEF level of Pr^3+^ is well known to be located at 4 meV above the ground state (Goossens *et al.*, 1996 ▸; Andreeff *et al.*, 1978 ▸). Figs. 2 ▸(*a*)–2 ▸(*c*) show the reconstructed scattering function *S*(*t*_1_, *t*) in terms of the incident TOF *t*_1_ *versus* the total TOF *t*, obtained using equation (4) on neutron data events collected on the detectors at scattering angles of 2θ = 16° and 2θ = 145°, and summation over all detectors. In all cases, the spectra show the dominant elastic line and a weak inelastic scattering line on the energy-loss side. Converting the data from the time domain to the energy domain clearly identifies the inelastic peak as the CEF excitation of Pr^3+^ near 4 meV (Fig. 2 ▸ *d*). The energy resolution as estimated from the FWHM of the elastic line ranges from 0.4 to 2.5 meV for neutrons with incident energies of *E*_i_ = 12–50 meV, respectively. These values are comparable to the energy resolution obtained with traditional instrumentation used to obtain energy discrimination, namely triple-axis and direct TOF spectrometers, but these do not provide efficient coverage over large volumes of reciprocal space. The present measurements therefore demonstrate that the CORELLI spectrometer is capable of achieving the desired degree of energy discrimination, with Δ*E*/*E*_i_ = 3–5% for the incident energy range mentioned above. However, we note that the reconstruction of the scattering function *S*(*t*_1_, *t*) at a fixed total TOF *t* involves all of the measured intensities at different correlation chopper phases *t*_0_ [equation (2)]. Therefore, the statistical error in *S*(*t*_1_, *t*) is approximately the same for all *t*_1_ and is proportional to the average signal [over *t*_1_ in *S*(*t*_1_, *t*) for constant *t*] containing both static and dynamic channels. This has led to the statement that the cross-correlation technique is ineffective for weak signals. But to reiterate, for the case of cross correlation on a direct TOF instrument at a pulsed source as considered here, the error bars come from the average spectrum *S*(*t*_1_, *t*) for constant *t* which contains only one single elastic channel. Diffuse scattering is generally considered to be ‘weak’ when compared with the Bragg peak intensities. But for a measurement performed at an arbitrary momentum transfer *Q*, the elastic diffuse scattering is the only elastic signal in *S*(*t*_1_, *t*) (for fixed *t*) and is likely to be strong (or at least similar) compared with the phonons or other inelastic contributions we want to eliminate. The cross correlation therefore provides an efficient method for measuring elastic diffuse scattering. These variance effects are clearly observed in Fig. 2 ▸(*d*), where the background of the reconstructed scattering function fluctuates strongly about zero owing to statistical effects.

**Figure 3:**
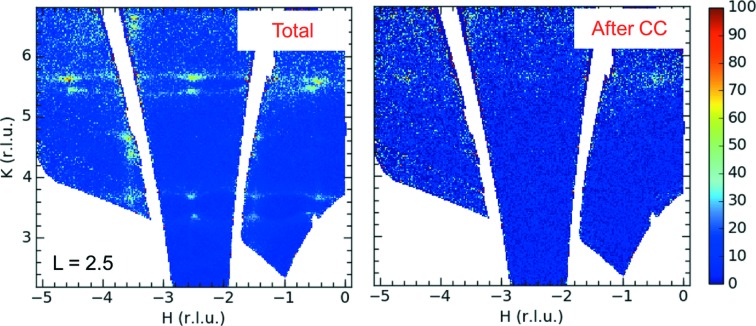
A single measurement on CORELLI generates two data sets, a total neutron scattering data set (left) and an elastic-only neutron scattering data set (right). The data shown here were collected from an SrTiO_3_ single crystal, showing the reciprocal-lattice plane (*H*, *K*, 2.5), with the data integrated in the normal direction over a range of 0.10 reciprocal lattice units (r.l.u.).

Measuring the diffuse scattering from a single crystal provides significant advantage over a powder diffraction experiment, since it provides a direct reference frame from which the orientational information of the diffuse scattering is obtained. This allows accurate three-dimensional reconstruction of the complex disorder if sufficiently complete scattering data sets are available. In most instances, it is necessary to include all symmetry-allowed orientations of the extended defect for modelling the data. The information from single-crystal samples is much richer than that from polycrystalline samples, liquids or amorphous materials, where the spherical averaging of the pair–pair correlation function is inherent. A typical single-crystal scattering experiment on CORELLI generates two data sets: one containing the total scattering data, comprising elastic as well as all inelastic events, and the elastic-only neutron scattering data set. Fig. 3 ▸ shows a part of the two data sets, the (*H*, *K*, 2.5) planes obtained for an SrTiO_3_ single crystal at 300 K at a single fixed crystal orientation.

SrTiO_3_ is a prototypical crystal to test the soft-mode theory of structural phase transitions, showing a cubic to tetragonal phase transition at *T*_c_ ≃ 105 K (Shirane *et al.*, 1993 ▸; Shirane & Yamada, 1969 ▸). A characteristic feature of this transition is the zone-boundary phonon instability, where the phonon softens towards zero energy as the temperature decreases towards *T*_c_ from above. The temperature and *Q* dependence of this soft mode have been characterized with an excitation energy of 6.8 meV at the *R* point at 300 K (Shirane *et al.*, 1993 ▸). By comparing the total and elastic-only scattering data sets, the inelastic origin of the split lines observed in the total scattering data set is evident (Fig. 3 ▸). It is almost entirely removed from the elastic-only data set after cross-correlation analysis. There remains a weak diffuse signal above the background at higher absolute *Q* regions, *e.g.* along *K* ≃ 5.5 reciprocal-lattice units (r.l.u.) in the elastic channel, which is due to the worsening energy resolution for higher-incident-energy neutrons, allowing more and more inelastic intensity to leak into the elastic channel. This illustrates one complication in the implementation of the cross-correlation technique at CORELLI for energy discrimination, as the energy resolution varies with the absolute value, *Q*, of the momentum transfer, which varies with incident neutron energy. But these data clearly illustrate the importance of energy discrimination for the determination of the origin of diffuse scattering features.

CORELLI currently has a solid-angle detector coverage of 2 steradian, which can be further expanded to 2.4 steradian when all planned 91 detector modules are installed. The wide wavelength band, high neutron flux and large detector coverage enable CORELLI to perform single-crystal neutron diffuse scattering experiments at an unprecedented data collection rate (Welberry & Whitfield, 2018 ▸). A full single-crystal diffuse scattering data set is generally collected at different angles by rotating the sample with a typical step size between 2 and 5°. Using the *MANTID* software (Arnold *et al.*, 2014 ▸), the collected data are transformed into reciprocal space and merged together to generate a three-dimensional data set in reciprocal space for next-step data analysis.

**Figure 4:**
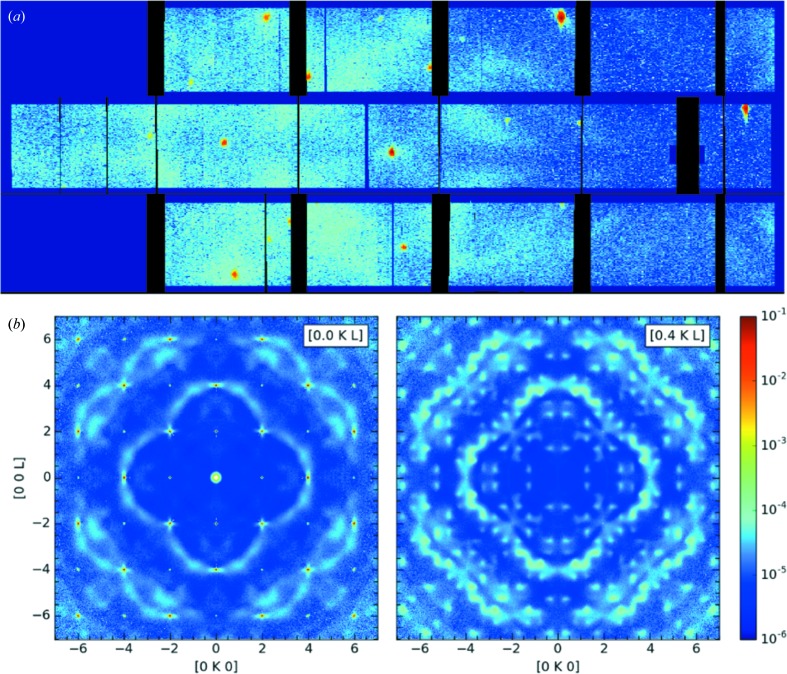
Elastic diffuse neutron scattering data collected from a Zr_0.85_Ca_0.15_O_1.85_ single crystal on CORELLI at room temperature. (*a*) Instrument view of the diffraction data for one crystal orientation. (*b*) Slices of the reciprocal-lattice planes (0, *K*, *L*) and (0.4, *K*, *L*) with the data integrated in the normal direction over a range of 0.1 r.l.u. The data were collected at 91 angles with a step size of 2° and then merged after cross correlation and symmetrization using the *mmm* Laue point group.

Fig. 4 ▸(*a*) shows a detector view of a representative experimental data set at a single angle collected from a calcia-stabilized zirconia single crystal, Zr_0.85_Ca_0.15_O_1.85_, at room temperature, showing the coexistence of diffuse and Bragg scattering components. Ca-doped ZrO_2_ is an ionic conductor, where the Ca doping induces oxygen vacancies and gives rise to high oxygen ion mobility. Rather than being randomly distributed throughout the lattice, the introduced oxygen vacancies form short-range-ordered structures, which give rise to highly structured diffuse scattering patterns in reciprocal space. Both the Bragg peaks and diffuse scattering can be easily seen from the detector view. Fig. 4 ▸(*b*) shows two representative slices of the elastic data in the (0, *K*, *L*) and (0.4, *K*, *L*) planes, respectively. The sharp red spots are Bragg peaks and the light-blue features are diffuse scattering. In contrast with the Bragg peaks which are sharply isolated in reciprocal space, the diffuse scattering patterns are distributed broadly over reciprocal space. The neutron diffuse scattering technique is very effective for characterizing the oxygen vacancy correlation because the neutron cross section of light oxygen ions is comparable to those of the heavier elements. In fact, the observed diffuse scattering here is mainly caused by oxygen vacancies. Two main types of local structure were reported in earlier studies (Osborn *et al.*, 1986 ▸; Andersen *et al.*, 1986 ▸; Proffen & Welberry, 1998 ▸): (i) individual oxygen vacancies with relaxed neighbouring ions, and (ii) oxygen vacancy pairs separated by 3^1/2^/2*a* along the [111] direction. With CORELLI, a full neutron elastic single-crystal diffuse scattering data set can be collected in less than a day, much shorter than the time that would be needed to obtain the same coverage and statistics on a traditional chopper spectrometer with a monochromatic incident beam.

The energy discrimination achieved through cross correlation and the high efficiency of the white-beam Laue technique to provide coverage of large volumes of reciprocal space on CORELLI allow quantitative measurement of the elastic-only diffuse scattering and address a wide range of scientific problems. These include diffuse scattering arising from either atomic disorder or static displacements in solid-solution alloys where the properties are intimately connected to the microstructure (Ice & Sparks, 1999 ▸; Nield & Keen, 2001 ▸; Kostorz, 1996 ▸; Schweika, 1998 ▸). Another area of interest is complex and disordered molecular crystals, both small ones including anthracene and benzene derivatives, and large-scale molecules like proteins and polymers, which typically consist of comparatively rigid molecules that are weakly bound. A common theme in these molecular systems is orientational disorder, giving rise to short-range correlation (Welberry, 2005 ▸). The diffuse scattering from these systems contains information on both the molecule itself and the intermolecular orientational and dynamic correlations, thus providing important characterization that relates to the mean molecular structure and the intermolecular mobility (Westhof *et al.*, 1984 ▸). Short-range spin fluctuations in magnetically frustrated systems constitute another field attracting substantial interest (Ramirez, 1994 ▸). For example, the spin ice system has highly degenerate ground states with non-vanishing entropy, even when the temperature approaches absolute zero. Local constraints may lead to short-range ordered spin structures, which give rise to characteristic diffuse scattering patterns (Fennell *et al.*, 2009 ▸; Morris *et al.*, 2009 ▸). Such systems can be described by well defined microscopic Hamiltonians that allow the development of theoretical frameworks to interpret the experimental observations. The close relationship between theory and experiment further prompts the exploration of emerging phenomena with external perturbations, including magnetic fields, which are well suited for magnetic diffuse scattering studies (Kadowaki *et al.*, 2009 ▸). Complementary to the polarization analysis mentioned earlier, such studies over large regions of reciprocal space provide important characterizations of magnetically frustrated systems.

Finally, we point out that, although analysis of the information-rich diffuse scattering data remains intellectually challenging, there have been greater advances characterizing the diffuse scattering for complex and dis­ordered materials with enhanced computational power and reduced cost. It has been illustrated that the combination of graph theoretical and symmetry aspects with molecular modelling (Bürgi *et al.*, 1992 ▸), genetic evolution algorithms along with Monte Carlo simulation (Weber & Bürgi, 2002 ▸) and massive high-performance computing (Sudholt *et al.*, 2005 ▸) offer the potential of bringing about a paradigm shift in the structure determination and optimization of disordered materials. Theoretical developments like three-dimensional PDF analysis have also set the framework for characterizing the local structure of disordered materials in single-crystal form (Weber & Simonov, 2012 ▸).

In summary, the implementation of the cross-correlation technique makes CORELLI a dedicated highly efficient elastic diffuse scattering instrument. The high neutron flux and wide angular detector coverage at the instrument provide a unique capability to capture the atomic structure of dis­ordered crystalline and bulk materials structures *via* single-crystal diffuse scattering methods.
